# Toxic Effects of 3,3′-Iminodipropionitrile on Vestibular System in Adult C57BL/6J Mice *In Vivo*

**DOI:** 10.1155/2020/1823454

**Published:** 2020-07-03

**Authors:** Shan Zeng, Wenli Ni, Hui Jiang, Dan You, Jinghan Wang, Xiaoling Lu, Liman Liu, Huiqian Yu, Jingfang Wu, Fangyi Chen, Huawei Li, Yunfeng Wang, Yan Chen, Wenyan Li

**Affiliations:** ^1^ENT Institute and Otorhinolaryngology Department of Affiliated Eye and ENT Hospital, State Key Laboratory of Medical Neurobiology, Fudan University, Shanghai 200031, China; ^2^Institutes of Biomedical Sciences, Fudan University, Shanghai 200032, China; ^3^The First Affiliated Hospital of Sun Yat-sen University, Guangzhou 510080, China; ^4^Department of Biomedical Engineering, Southern University of Science and Technology, Shenzhen, Guangdong 518055, China

## Abstract

The utricle is one of the five sensory organs in the mammalian vestibular system, and while the utricle has a limited ability to repair itself, this is not sufficient for the recovery of vestibular function after hair cell (HC) loss induced by ototoxic drugs. In order to further explore the possible self-recovery mechanism of the adult mouse vestibular system, we established a reliable utricle epithelium injury model for studying the regeneration of HCs and examined the toxic effects of 3,3′-iminodiproprionitrile (IDPN) on the utricle *in vivo* in C57BL/6J mice, which is one of the most commonly used strains in inner ear research. This work focused on the epithelial cell loss, vestibular dysfunction, and spontaneous cell regeneration after IDPN administration. HC loss and supporting cell (SC) loss after IDPN treatment was dose-dependent and resulted in dysfunction of the vestibular system, as indicated by the swim test and the rotating vestibular ocular reflex (VOR) test. EdU-positive SCs were observed only in severely injured utricles wherein above 47% SCs were dead. No EdU-positive HCs were observed in either control or injured utricles. RT-qPCR showed transient upregulation of *Hes5* and *Hey1* and fluctuating upregulation of *Axin2* and *β-catenin* after IDPN administration. We conclude that a single intraperitoneal injection of IDPN is a practical way to establish an injured utricle model in adult C57BL/6J mice *in vivo*. We observed activation of Notch and Wnt signaling during the limited spontaneous HC regeneration after vestibular sensory epithelium damage, and such signaling might act as the promoting factors for tissue self-repair in the inner ear.

## 1. Introduction

The sensory organs of the mammalian inner ear include the organ of Corti in the cochlea, which senses sound, and the macula sacculi, macula utriculi, and crista ampullaris from the vestibular system that sense acceleration and postural signals. HCs are the mechanoreceptors in all inner ear sensory organs and are thus responsible for normal auditory and balance functions. HCs are vulnerable to multiple injury factors, including genetic abnormalities, aging, noise, infection, ototoxic drugs, traumas, and tumors [1, 2]. Unlike nonmammalian vertebrates, the sensory organs of the mammalian inner ear have only very limited self-renewal capacity in the vestibular system and no self-renewal in the cochlea, and this means that dysfunction of vestibular sensation and hearing is permanent [1–3]. Thus, it is a very important task to promote HC regeneration and rebuild the function of the inner ear. However, unlike a cochlear injury model, there are few practical vestibular injury models reported with data of objective tests for evaluating the function of the vestibular system in mice similar to the ABR test for hearing or the changes of gene expression. The present work focused on the development of the IDPN-induced adult mouse vestibular injury model.

The compound 3,3′-iminodiproprionitrile (IDPN) has been reported to be neurotoxic and vestibulotoxic [4, 5] resulting in loss of vestibular sensory epithelial cells in rats and mice and leading to irreversible loss of peripheral vestibular function [6–10], and thus, IDPN can be used for an *in vivo* model of adult mammalian vestibular dysfunction. In the present study, we further evaluated the dose-dependent toxic effects of IDPN on the vestibular epithelium in adult C57BL/6J mice, and the rotating vestibular ocular reflex (VOR) was measured as an objective reflection of the function of the vestibular system, which was crossvalidated with the traditionally used swim test [10–12]. Furthermore, we investigated the limited self-renewal process in the mouse utricle after the injury induced by a single injection of IDPN, as well as the gene expression profile related to multiple signaling pathways during the HC regeneration process, which might provide the potential signaling targets for promoting the HC regeneration.

## 2. Materials and Methods

### 2.1. Animals

Adult C57BL/6J wild-type mice 30 days old (P30) and weighing about 20 g were provided by the Department of Laboratory Animal Science of Fudan University. Each animal in the acute injury group received a single intraperitoneal injection of IDPN (TCI Shanghai, No. I0010, 2, 3, 4, 5, or 6 mg IDPN/g body weight, *d* = 1.02 g/mL, 1 *μ*L ≈ 1 mg) or saline (0.9% *w*/*v* NaCl) regardless of their gender or estrous cycle stages (*n* = 3 animals for each group). Each animal in the subacute injury group received a daily intraperitoneal injection of IDPN (0.5, 0.75, or 1 mg IDPN/g body weight) or saline (0.9% *w*/*v* NaCl) for 7 consecutive days (*n* = 3 animals for each group). To explore if there was spontaneous cell regeneration, EdU was intraperitoneally injected daily at 5 mg/mL starting at D6 after IDPN injection (*n* = 3 animals for each group). All animal experiments were approved by the Institutional Animal Care and Use Committee of Fudan University.

### 2.2. Vestibular Function Tests

The swim test was evaluated on day 7 (D7) after IDPN injection (0, 2, 4, and 6 mg IDPN/g body weight, *n* = 3 animals for each group). The mouse was placed in a standard cage with about 30 cm of warm water (about 37°C) in it. Swimming was recorded by camera and scored 0–3 according to their swim behavior [11]. Vestibular function was also evaluated by a binocular VOG-based VFT system provided by Prof. Fangyi Chen's team from Southern University of Science and Technology at 7 days (7 d), 1 month (1 m), and 3 months (3 m) after IDPN injection (0, 2, 4, and 6 mg IDPN/g body weight, *n* = 3 animals for each group). IDPN-administrated mouse was placed in a custom-built box adapted to its weight and then fixed on the rotating platform. Mirror images of eye movement were synchronously recorded by the side cameras as the platform rotated at 0.25, 0.5, and 1 Hz under infrared illumination. The recording frame rate was 30 fps, and each record contained at least 1000 frames. Videos of the mouse's pupil movements were then analyzed by customized software to acquire pupil position data. Exported eye-location data underwent Fourier transformation using the MATLAB 2016b software to obtain amplitude data for eye movement, then calculated to gain values, which are defined as the ratio of amplitude between response and stimulus [12].

### 2.3. Histological Labeling

Temporal bones were dissected after the animals were sacrificed by cervical dislocation. The utricles were harvested under a stereomicroscope then fixed in 4% paraformaldehyde (Sigma) for 20 min at 4°C, rinsed in PBS, and decalcified in 10% EDTA for 5–10 min at 37°C to remove the otolith. All utricles were blocked with 10% goat serum and 1% Triton X-100 in PBS overnight at 4°C. All antibodies were diluted in 1% Triton X-100 in PBS. Primary antibodies included rabbit anti-MyosinVIIa (anti-MyoVIIa, 1 : 800 dilutions, Proteus BioSciences, No.20-6790) to mark HCs and goat anti-Sox2 (1 : 300 dilutions, Santa Cruz, No.sc-17320) to mark Sox2+ cells. Alexa Fluor 647 Donkey anti-Rabbit (1 : 500 dilutions, Invitrogen, No. A-31573) and Alexa Fluor cy3 Donkey anti-Goat (1 : 500, dilutions, Jackson, No.705-165-003) were used for detection of primary antibodies. The Click-iT EdU Imaging Kit (Life Technologies, No. E10415) was used to identify proliferative cells. Nuclei were labeled with DAPI (1 : 1,000 dilutions, Invitrogen, No. D3571).

### 2.4. Cell Counts in the Utricle

Stained utricles were scanned with a confocal microscope (Leica TCS SP8, 40x oil objective, resolution 1024 × 1024, slice thickness 1–1.5 *μ*m). The numbers of MyoVIIa+ cells (HCs), Sox2+-supporting cells (SCs), and Sox2+/MyoVIIa+ cells (Sox2+ HCs) in the striolar and extrastriolar regions were counted with ImageJ software per 100 *μ*m × 100 *μ*m area; two different areas were counted in the striolar and extrastriolar regions, for every utricle.

### 2.5. RNA Extraction and Real-Time PCR

TRIzol (Life Technologies, No.15596-026) was used to isolate total RNA, and mRNA was reverse transcribed with the GoScript Reverse Transcription System (GoTaq 2-Step RT qPCR System, Promega, No. A6010) according to the manufacturer's protocol. Utricle samples were dissected in cold PBS at 4°C and put into the TRIzol solution immediately and the RNA isolation was processed. Isolated RNA samples were preserved at -20°C. Quantitative real-time PCR was performed using the GoTaq qPCR Master Mix (GoTaq 2-Step RT qPCR System, Promega, No. A6010) and analyzed on an Applied Biosystems 7500. Samples were placed on ice throughout the experiment. The required cDNAs for each experiment were reversed from 1 *μ*g RNA. All primers were provided by the Invitrogen company (see Table 1) and *Gapdh* was used as the internal reference. Three repetitions were performed in each experiment, and two batches of samples were repeated for each group. The 2 − ΔΔCT method was used to analyze gene expression relative to *Gapdh* expression. Two-tailed Student's *t*-test was used to determine differences between groups.

### 2.6. Data Analysis

One-way or two-way ANOVA test and Tukey's multiple comparison test were employed for statistical analysis by GraphPad Prism 6.0. The detected difference was defined to be significant when *p* < 0.05. According to the acquired *p* values, three levels of significance (*p* < 0.05, *p* < 0.01, and *p* < 0.001) were rated and indicated in the results.

## 3. Results

### 3.1. A Single Injection of IDPN Caused Dose-Dependent Acute Utricular Sensory Epithelium Injury

**Figure 1:**
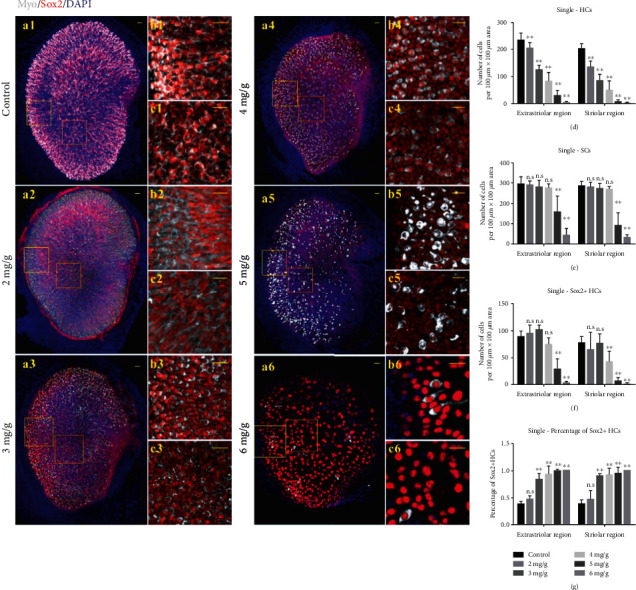
A single injection of IDPN induced dose-dependent acute utricular sensory epithelium injury. The numbers of HCs, SCs, and Sox2+ HCs were counted with ImageJ software per 100 *μ*m × 100 *μ*m area, two different areas in the extrastriolar region (yellow square frame) and striolar region (orange square frame) for every utricle (a1–a6). (b1–c6) showed zoomed pictures within the yellow (b1–b6) and orange (c1–c6) square rectangle tool. In controls (a1), MyoVIIa+ HCs, and Sox2+ SCs were arranged in an orderly fashion and showed no defects. MyoVIIa+ HCs became sparser dose-dependently, especially in the striolar area (b2–b6, c2–c6). No significant loss of Sox2+ SCs was observed until 5 or 6 mg IDPN/g body weight was injected (b2–b6, c2–c6). Graphs (d, e, f) show the average numbers of HCs, SCs, and Sox2+ HCs, in the extrastriolar and striolar regions per 100 *μ*m × 100 *μ*m area after a single IDPN injection. Graph (g) shows the percentage of Sox2+ HCs after a single IDPN injection. The scale bars indicate 20 *μ*m. ^∗∗^*p* < 0.01.

In order to obtain a reliable adult mouse utricular injury model *in vivo*, we gave adult mice (P30) a single intraperitoneal injection of IDPN at different doses (2, 3, 4, 5, and 6 mg/g body weight). Compared with controls, the mice started to show minor abnormal behavior like head tossing/bobbing at D4 after IDPN injection, while no obvious sensory cell loss was observed in the utricle by immunostaining at this stage (data not shown). Vestibular dysfunction increased gradually until reaching a stable state at D6, and the utricles were harvested at D7. In the saline controls, HCs and SCs were arranged in an orderly fashion with no defects (Figures 1(a1)–1(c1)). The density of HCs decreased dose-dependently as the IDPN concentration increased from 2 to 6 mg/g body weight, especially in the striolar area (Figures 1(a1)–1(c6)). At 2, 3, 4, 5, and 6 mg IDPN/g body weight, the HCs were about 66.82 ± 11.03% and 87.01 ± 9.69%, 42.26 ± 10.69% and 52.84 ± 7.06%, 24.52 ± 16.51% and 35.45 ± 13.83%, 3.41 ± 3.11% and 12.56 ± 8.30%, and 0.12 ± 0.31% and 1.03 ± 1.21% that of controls in the striolar and extrastriolar areas, respectively. Total HC loss at each IDPN concentration in both the striolar and extrastriolar areas was significant compared with control group, respectively (Figure 1(d), *p* < 0.01). There was no SC loss until the IDPN concentration reached 5 mg/g body weight (Figure 1(e), *p* < 0.001). In contrast, no significant loss of Sox2+ HCs, which are assumed to be Type II HCs [13], was observed in both the extrastriolar and striolar regions of the 2 and 3 mg IDPN/g body weight mice (Figure 1(f), *p* > 0.05). The proportion of Sox2+ HCs among the HCs was 46.58 ± 6.71% in the extrastriolar area and 47.23 ± 15.57% in the striolar area when the IDPN dosage was 2 mg/g body weight, and this was similar to that of the control group (Figure 1(g), *p* > 0.05). As the IDPN dosage increased from 3 to 6 mg/g body weight, the proportion of Sox2+ HCs among the HCs also increased from 83.26 ± 11.04% in the extrastriolar area and 89.67 ± 3.70% in the striolar area to 100% in both areas (Figure 1(g), *p* < 0.001).

### 3.2. Consecutive Injection of IDPN Caused Dose-Dependent Subacute Utricular Sensory Epithelium Injury

**Figure 2:**
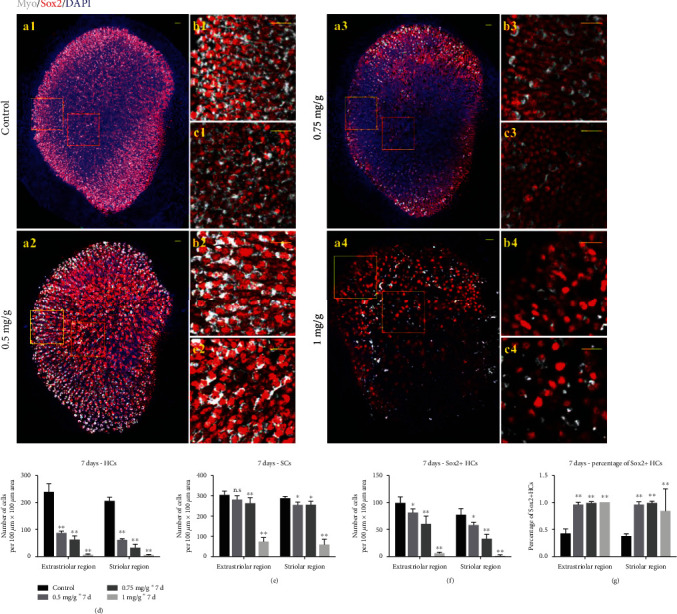
Consecutive injection of IDPN induced dose-dependent subacute utricular sensory epithelium injury. In controls (a1), MyoVIIa+ HCs and Sox2+ SCs were arranged in an orderly fashion and had no defects. MyoVIIa+ HCs also became sparser dose-dependently especially in the striolar area (b2–b4, c2–c4). A slight decrease in Sox2+ SCs can be seen in the utricle after 7 consecutive days of 0.5 and 0.75 mg IDPN/g body weight injection (c2–c3). Obvious loss of HCs and SCs was observed in the utricles when 1 mg/g body weight IDPN was consecutively administered, and only a few HCs survived (a4–c4). Graphs (d, e, f) showed the average numbers of HCs, Sox2+ SCs, and Sox2+ HCs, in the extrastriolar and striolar regions per 100 *μ*m × 100 *μ*m area after 7 consecutive days of IDPN injection. Graph (g) shows the percentage of Sox2+ HCs after 7 consecutive days of IDPN injection. The scale bars indicate 20 *μ*m. ^∗^*p* < 0.05 and ^∗∗^*p* < 0.01.

P30 mice received daily intraperitoneal injections of IDPN (0.5, 0.75, or 1 mg/g body weight) or saline (0.9% *w*/*v* NaCl) for 7 consecutive days. The mice showed head tossing/bobbing starting at D7, and utricles were harvested at D14 (7 days after the last injection). In the control group, HCs and SCs were arranged in an orderly fashion and showed no obvious defects (Figures 2(a1)–2(c1)). The density of HCs decreased dose-dependently as the IDPN concentration increased from 0.5 to 1 mg/g body weight, especially in the striolar area (Figures 2(a2))–2(a4), 2(b2)–2(b4), 2(c2)–2(c4), and 2(d)). Obvious SC loss was observed in the utricles when 1 mg/g body weight IDPN was consecutively administered, and very few HCs survived in these samples (Figures 2(b2)–2(b4), 2(c2)–2(c4), and 2(e)). More than 90% of the surviving utricular HCs were Sox2+ HCs (Type II HCs) after consecutive injections of IDPN (Figure 2(g)).

There was no significant difference between the injury induced by either the single 4 mg/g body weight IDPN injection or the consecutive 0.5 mg/g body weight IDPN injections except SCs in the striolar area (the cumulative IDPN concentration was 3.5 mg/g body weight) (HCs in the extrastriolar area: 83.25 ± 32.48 vs. 85.50 ± 7.61, *p* = 0.87; HCs in the striolar area: 49.75 ± 33.49 vs. 59.67 ± 5.09, *p* = 0.49; SCs in the extrastriolar area: 278.42 ± 16.45 vs. 281.17 ± 15.16, *p* = 0.74; SCs in the striolar area: 269.33 ± 13.57 vs. 252.17 ± 16.31, *p* = 0.03). There was greater HC loss and Sox2+ SC loss caused by the single IDPN injection when we compared the samples between the cumulative 0.75 mg IDPN/g body weight injections and the single injection of 5 mg IDPN/g body weight (HCs in the extrastriolar area: 60.5 ± 14.8 vs. 29.50 ± 19.50, *p* = 0.004; HCs in the striolar area: 31.67 ± 10.09 vs. 6.92 ± 6.32, *p* ≤ 0.0001; SCs in the extrastriolar area: 259.83 ± 25.83 vs. 157.58 ± 79.68, *p* = 0.008; SCs in the striolar area: 254.33 ± 17.24 vs. 94.42 ± 58.75, *p* < 0.0001). Based on these results, a single IDPN injection may cause more severe vestibular damage as the IDPN concentration increases.

### 3.3. Rotating VOR Test Served as a More Sensitive Objective Measurement for the Function of the Vestibular System

Behavioral tests and electrophysiological examination are commonly used methods for vestibular assessment for mice [11, 14, 15]. However, it is very difficult to test the function of the utricle exactly and directly without surgery. Behavior observation, swim test, and rotating VOR test were employed to evaluate the overall vestibular function after IDPN administration.

**Figure 3:**
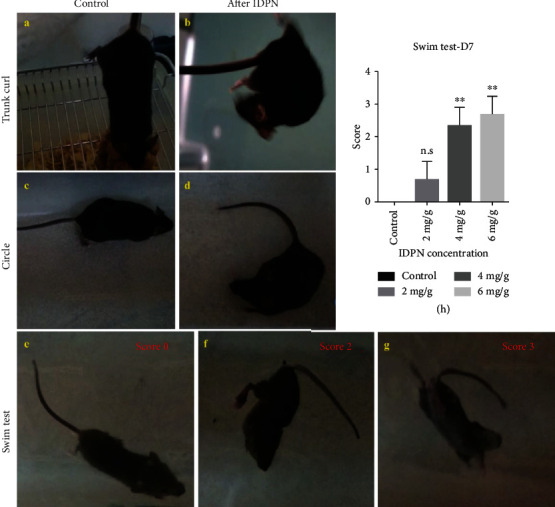
The behavioral tests showed the dysfunction of the vestibular system after IDPN injection. A simple trunk curl test showed that control mice would reach towards the horizontal surface when their tails were held (a), while the mice with vestibular injury would curl towards their abdomen (b). When placed on a table, control mice would walk straight and balanced (c), while animals with vestibular impairment would walk in circles chasing their tails (d). The swim test can yield a score for general vestibular function. Mice in water will swim (e) (score 0), swim irregularly (not shown in figure, score 1), float immobile (f) (score 2), or tumble underwater (g) (score 3). Graph (h) showed the swim test scores on D7 after different concentrations of single IDPN injections which get higher with the increase in IDPN dose. However, scores showed no significant difference in the 2 mg IDPN/g body weight injected mice compared to controls. Ordinary one-way ANOVA of swim test (*p* = 0.0005) with Tukey's multiple comparisons test were performed with GraphPad Prism 6.01. ^∗∗^*p* < 0.01.

Because dysfunction of the vestibular system generally manifests as disequilibration or disorientation, we observed the mice's behavior daily after IDPN injection to evaluate the function of the vestibular system [11]. The mice showed spontaneous head shaking at D4 after a single IDPN injection, and the severity of head shaking was positively correlated with the dose of IDPN. Furthermore, the mice showed trunk curling (Figures 3(a) and 3(b)) and circling (Figures 3(c) and 3(d)) at D6 after a single injection of 4 mg IDPN/g body weight.

The swim test was performed at D7 to quantitatively evaluate the effect of IDPN on vestibular function [11]. Mice from the control group performed normal swimming behavior in which the tail propelled their movement through the water, and they balanced their heads and backs on the water with all four limbs in motion (Figure 3(e), score 0). Mice from the IDPN injection group displayed vestibular anomalies in the water. Mouse displayed normal swimming (score 0) or slightly unbalanced swimming behavior (score 1) after 2 mg/g body weight IDPN administration. When the dose of IDPN reached 4 mg/g body weight or more, the swim scores were all 2 or 3 (Figures 3(f) and 3(g)). Scores showed no difference after 2 mg/g body weight IDPN injection (Figure 3(h), average score 0.67 ± 0.47, *p* > 0.05 compared with control mice) and were significantly raised when the IDPN dose increased from 4 to 6 mg/g body weight (Figure 3(h), average score 2.33 ± 0.47 and 2.67 ± 0.47, respectively, all *p* < 0.01 compared with the control mice). There were no significant differences between the 4 and 6 mg IDPN/g body weight groups (Figure 3(h), *p* > 0.05).

**Figure 4:**
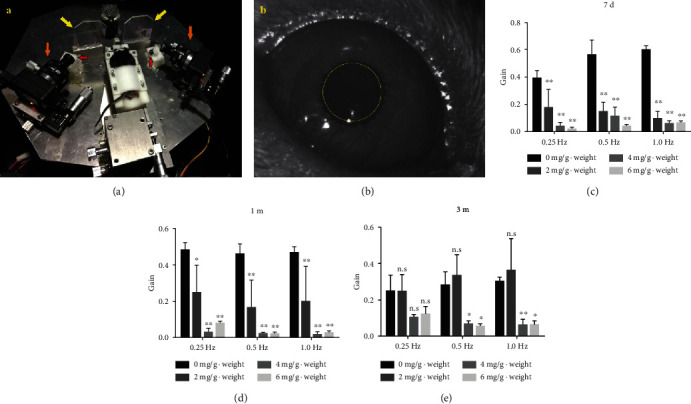
Rotating VOR test served as a sensitive measurement for evaluating the function of the vestibular system. Vestibular function was evaluated on a binocular VOG-based VFT system at 7 days (7 d), 1 month (1 m), and 3 months (3 m) after IDPN injection. A mouse was placed in the custom-built box adapted to its weight and then fixed on the rotating device (a). Yellow arrows, orange arrows, and red arrows point out the mirrors, cameras, and infrared lamps on the platform, respectively (a). Eye movements in the front mirror were recorded in real time by the side cameras as the platform rotated at 0.25, 0.5, and 1 Hz (b). Graphs (c, d, e) show the rotating VOR gain at 7 d, 1 m, and 3 m. VOR gain showed significant decrease at all three frequencies compared with control mice dose-dependently in day 7 (c) and 1 month (d). VOR gains of the 2 mg IDPN/g body weight injected mice showed no difference compared with those of the control mice after 3 months (e). Two-way ANOVA of VOR gains (all *p* < 0.0001) with Tukey's multiple comparison test was performed with GraphPad Prism 6.01. ^∗^*p* < 0.05 and ^∗∗^*p* < 0.01.

Dysfunction of the vestibular system also manifests as the interruption of rotating VOR, which is generally important for dynamic visual acuity, which is one essential vestibular function. Thus, we used a binocular VOG-based VFT system and customized software to quantitatively evaluate the vestibular function which served as an objective index for vestibular function. The rotating VOR test was performed at D7 after a single dose of IDPN or saline injection. The mouse was placed in a custom-built box adapted to its weight and then fixed on the rotating device (Figure 4(a)). Eye movements in the front mirror were recorded in real time by the side cameras as the platform rotated at 0.25, 0.5, and 1 Hz (Figure 4(b)).

Our results showed that the pixels of eye movement amplitude of control mice were 31.75 ± 4.65, 46.00 ± 8.41, and 48.57 ± 2.42 at 0.25, 0.5, and 1 Hz, respectively (*n* = 3). When we evaluated the VOR test 7 days after 2 mg/g body weight IDPN injection, eye movement amplitude dropped to 14.33 ± 11.01, 11.94 ± 5.46, and 7.54 ± 4.67 at 0.25, and 0.5 Hz, respectively (*n* = 3). VOR gain showed significant decrease (Figure 4(c), *p* < 0.01 all three frequencies, compared with the control mice), which was more sensitive with vestibular dysfunction compared to the results of the swim test. There are no significant differences between 4 and 6 mg/g body weight IDPN injection groups (Figure 4(c), *p* > 0.05) since the eye movement amplitude dropped to 3.19 ± 2.50, 9.24 ± 5.52, and 4.71 ± 1.64 and 1.60 ± 0.98, 3.02 ± 0.63, and 5.12 ± 0.89 at 0.25, 0.5, and 1 Hz, respectively. To observe the vestibular function recovery or compensation, we performed rotating VOR test at 1 month and 3 months. Only two of three mice in the 6 mg/g body weight IDPN-injected group survived both 1 month and 3 months after IDPN injection (Figures 4(d) and 4(e)). VOR gain data stayed significantly decreased compared to that of the control group at 1 month (Figure 4(d)). Results showed that eye movement amplitude data almost recovered to normal (20.48 ± 7.08, 27.21 ± 9.32, and 29.41 ± 14.10 at 0.25, 0.5, and 1 Hz, respectively, in 2 mg/g body weight group, all *p* > 0.05) compared with those of the control mice after 3 months. No VOR gain recovery appeared in the 4 mg/g body weight group (Figure 4(e), all *p* > 0.05, compared with control mice after 3 months). These data may conclude that vestibular function recovery or compensation depends on the remaining hair cells and supporting cells.

### 3.4. Transient Upregulation of Notch and Fluctuating Upregulation of Wnt Signaling-Related Genes after IDPN Administration

**Figure 5:**
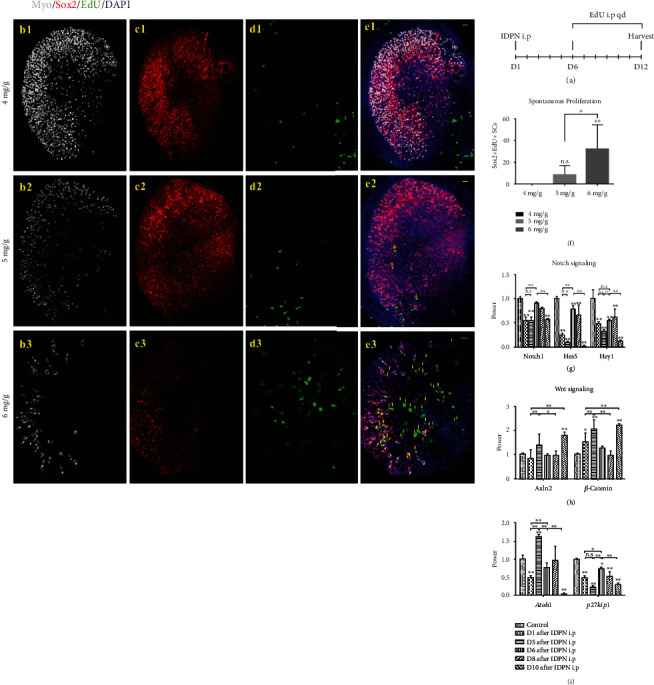
The limited proliferation in utricle sensory epithelium induced by IDPN injection. No MyoVIIa+/EdU+ cells were seen in any of the IDPN-treated utricles (c1–c3). EdU+/Sox2+ cells were not observed in the 4 mg IDPN/g body weight-treated utricles (e1). EdU+/Sox2+ cells appeared on the edge of the striolar area and the striolar area in the 5 and 6 mg IDPN/g body weight-treated utricles (yellow arrows) (e2, e3). Graph (f) shows the average number of EdU+/Sox2+ cells in the 4, 5, and 6 mg IDPN/g body weight-treated utricles. Graph (g) shows changes in Notch signaling genes at D1, D5, D6, D8, and D10 after 6 mg IDPN/g body weight injection. Graph (h) shows changes in Wnt signaling genes after IDPN injection. Graph (i) shows changes in *Atoh1* and *p27kip1* expression after IDPN injection. All hair cell development-related vital genes showed a fluctuating change. The scale bars indicate 20 *μ*m; ^∗^*p* < 0.05 and ^∗∗^*p* < 0.01.

It has been reported that the utricle has a limited ability to repair itself after HC loss [16]. We observed a slight recovery of vestibular function according to the rotating VOR test results at 3 months after 2 mg IDPN/g body weight injection. To further evaluate the spontaneous regeneration of HCs and SCs in the utricle after HC loss induced by IDPN injection, we harvested the utricles at D12 after IDPN injection. In order to label the new HCs that were generated through proliferation, EdU was intraperitoneally injected daily at 5 mg/mL starting at D6 after IDPN injection (Figure 5(a)). There were no EdU+ HCs in the utricles from either control (data not shown) or IDPN-injected mice (Figures 5(b1)–5(e3)). We only observed EdU+ SCs at the striolar region of the utricles from the mice injected with higher doses of IDPN (Figures 5(e2), 5(e3), and 5(f), 5 mg/g body weight IDPN: 8.67 ± 8.24, *p* > 0.05; 6 mg/g body weight IDPN: 31.67 ± 22.34, *p* < 0.01), while no EdU+ SCs were seen in the controls or at lower doses of the IDPN injection (4 mg IDPN/g body weight), which suggested that utricles do indeed have limited proliferative regeneration potential in response to severe injury and subsequent loss of epithelial integrity.

Based on the results above, we chose 6 mg IDPN/g body weight-treated utricles to explore the potential mechanism of spontaneous HC regeneration in the utricle. Utricles were harvested for RT-qPCR at D1, D5, D6, D8, and D10 after 6 mg IDPN/g body weight injection or normal saline injection. Total RNA was extracted for RT-qPCR to identify the genes involved in the Notch and Wnt signaling pathway in addition to the genes related to the processes of HC differentiation and maturation. Compared with controls, during the injury induced by IDPN injection and during the subsequent recovery process in the sensory epithelium of the utricle, *Notch1*, *Hey1*, and *Hes5* were downregulated during the whole process although with small fluctuations (Figure 5(g)). *Axin2*, *β-catenin*, and genes involved in the Wnt signaling pathway were upregulated (Figure 5(h)). The expression of *Atoh1*, a crucial gene for HC differentiation, was also upregulated in a similar manner to the genes involved in Wnt signaling. Meanwhile, *p27kip1* was downregulated, which was coincident with the increased proliferation of SCs during the recovery process of the damaged utricle (Figure 5(i)).

## 4. Discussion

Khan et al. reported that the brain and vestibule appear to be major target sites of IDPN, and the behavioral deficits that IDPN induced were reported to be identical to those of a bilateral labyrinthectomy [5]. Seoane et al. showed that extrusion is a major mechanism of HC death in mammals; that necrosis, apoptosis, and extrusion form a continuum of modes of HC loss; and that the intensity of the damaging stimulus determines the prevalence of each mode [17]. The association of HC degeneration with behavioral syndrome was also found in dose-response studies in acute, repeated, and chronic dosing in rats [6, 18, 19]. In the current study, IDPN was used to establish an utricle injury model in adult mice in an acute (one high dose) or a subacute (multiple small doses) manner. We found that similar dose-dependent utricle injury patterns were induced by IDPN in both the acute (single injection) and subacute (7 days of injection at lower concentrations) treatments in mice. Sensory cells in the striolar area are more vulnerable to IDPN than those in the extrastriolar area, and Type I HCs (MyoVIIa+/Sox2− cells) are more vulnerable to IDPN than Type II HCs (MyoVIIa+/Sox2+ cells), while both types of HCs are more vulnerable than SCs [8, 20]. The range of sensitivity to IDPN might be due to differences in cell structure as well as differences in the expression of Sox2. It has been reported that Type I HCs have significantly more stereocilia than Type II HCs, which might imply that Type I HCs have more mechanoelectric transduction channels for the entrance of IDPN [21, 22]. Oesterle et al. reported that Sox2 is absent from auditory HCs and Type I HCs in the utricle and that Sox2− HCs are more vulnerable to the aminoglycoside kanamycin, while Sox2+ HCs and SCs might not be affected by the same dose of the ototoxic drug. The resistance against IDPN seen in the Sox2+ cells might be related to the function of Sox2 for the maintenance of sensory cells in the adult mouse inner ear [13] or might be similar to the protective effect of the “nNOS-Sox2-Shh” pathway against ischemic neuronal damage and ischemia reperfusion injury [23, 24].

As is well known, HC loss in the utricle results in dysfunction of the vestibular system in mice, manifesting as spontaneous head shaking, trunk curling, walking in circles, and abnormal swimming behavior [11]. When the concentration of IDPN was 2 mg/g body weight, it could already cause about 12.99 ± 9.69% HC loss in the extrastriolar area and 33.18 ± 11.03% loss in the striolar area. At this dose of IDPN, the swim test was scored 0 to 1, which implied no significant dysfunction of the vestibular system. On the contrary, when we tested the mice with the rotating VOR, the eye movement amplitude and VOR gain dropped considerably at all three frequencies, which suggested that the rotating VOR test is more sensitive than the swim test for evaluating the function of the vestibular system. However, when HC loss reached above 35%, no differences were seen between the swim test and rotating VOR test.

At 3 months after injury, eye movement amplitude only recovered in the 2 mg IDPN/g body weight group where HC losses were not severe. In the 4 and 6 mg IDPN/g body weight groups, there could be seen only partial recovery at 0.25 Hz, which suggested that the recovery of vestibular function after long-term repair depended on the HCs and SCs that remained. There were no EdU+ HCs seen in either the control or IDPN-treated utricles, which means that the limited regeneration capacity in the utricles might only be the result of direct nonmitotic regeneration of HCs through SC transdifferentiation [3, 16, 25].

Wnt and Notch signaling have been reported to be vital for the fate determination of SCs and HCs during mammalian inner ear development [26–29]. The expression of CDK inhibitors like *p27kip1* can trigger the differentiation of precursor cells during auditory development [30, 31]. Wang et al. reported that a posttrauma decrease of *Hes5* expression and an increase in *Atoh1* expression might lead to the limited capacity for spontaneous HC regeneration [25]. To explore the potential mechanism of utricular self-regeneration, we investigated the expression of the Notch and Wnt signaling pathway genes *Atoh1* and *p27kip1* at D1, D5, D6, D8, and D10 after 6 mg IDPN/g body weight injection in which the greatest number of spontaneously self-regenerated SCs was seen. We observed decreased mRNA expression of *Notch1*, *Hey1*, and *Hes5* after the sensory epithelium injury induced by IDPN. The expression of these genes was partially upregulated at D6–D8 but then decreased again and was sustained at a relatively low level from D10. The expression of *Axin2* and *β-catenin*, important genes of the Wnt signaling pathway, showed an inverse expression pattern compared to that of the genes related to Notch signaling. The expression of *Atoh1* was consistent with the change in Wnt signaling pathway genes, while *p27kip1* expression changed in a similar manner as the Notch signaling genes. Slowik and Bermingham-McDonogh reported that Hes5 expression is maintained in some adult vestibular supporting cells [32, 33]. In this experiment, after IDPN administration, Notch signaling related genes and *p27kip1* experienced a transient decrease (D1-D5) which might be due to the cells undergoing IDPN striking. The subsequent transient activation of the Notch signal might be due to the activation of the Notch signal in the remaining cells after cell death. *Atoh1* experienced a very short downregulation (D1) then upregulated at D5. These fluctuating profiles of gene expression mimicked what is seen during inner ear development [26–29], which suggests that the limited proliferation of utricular SCs might be induced by the upregulation of Notch signaling and the *Atoh1* gene. The reported regeneration of HCs from SCs might be induced by the subsequent downregulation of Notch signaling and the *p27kip1* gene. Wnt/*β*-catenin signaling has an important role in protecting HCs against drug-induced HC loss [34] which might induced the transient upregulation after IDPN administration. Wnt signaling related genes then showed completely opposite changes with Notch signaling which might be because inhibition of Notch signaling could activate Wnt signaling [35]. It can thus be assumed that coregulating the genes involved in the HC regeneration process might enhance the recovery of the sensory epithelium and subsequent functional recovery.

In summary, we conclude that a single injection of IDPN is a convenient and reliable way to cause HC loss in adult mouse utricles and corresponding vestibular dysfunction *in vivo*. We also show that the rotating VOR test appears to be a more sensitive approach to evaluate the function of the vestibular system in a quantitative way. Finally, we conclude that it is likely that the limited HC self-regeneration ability in the adult mouse utricle includes the transient upregulation of Notch signaling and fluctuating upregulation of Wnt signaling.

## Figures and Tables

**Table 1:** RT-PCR primer sequence.

Gene	Forward primer (5′-3′)	Reverse primer (5′-3′)
p27kip1	CGGTGCCTTTAATTGGGTCT	AGCAGGTCGCTTCC TCATC
*β*-Catenin	ATGCGCTCCCCTCAGATGGTGTC	TCGCGGTGGTGAGA AAGGTTGTGC
Axin2	TGACTCTCCTTCCAGATCCCA	TGCCCACACTAGG CTGACA
Hes5	TGCTCAGTCCCAAGGAGAAA	AGCTTGGAGTTGG GCTGGT
Hey1	CACTGCAGGAGGGAAAGGTTAT	CCCCAAACTCC GATAGTCCAT
Notch1	GGAGGACCTCATCAACTCACA	CGTTCTTCAGGAG CACAACA
GAPDH	TGCGACTTCAACAGCAACTC	ATGAGGTCCACCACCCTGT

## Data Availability

The data statement has already been checked and are available from the corresponding author upon request.
